# Effects of Reminiscence Therapy on Cognition, Depression and Quality of Life in Elderly People with Alzheimer’s Disease: A Systematic Review of Randomized Controlled Trials

**DOI:** 10.3390/jcm11195752

**Published:** 2022-09-28

**Authors:** Davide Maria Cammisuli, Gabriele Cipriani, Emanuele Maria Giusti, Gianluca Castelnuovo

**Affiliations:** 1 Department of Psychology, Catholic University, 20123 Milan, Italy; 2 Division of Neurology, Versilia Hospital, 55049 Lido di Camaiore, Italy; 3 Istituto Auxologico Italiano IRCCS, Clinical Psychology Research Laboratory, 28824 Verbania, Italy

**Keywords:** reminiscence therapy, Alzheimer’s disease, aging, cognitive function, depression, quality of life

## Abstract

Background: Patients with Alzheimer’s disease (AD) present with cognitive function deterioration, neuropsychiatric symptoms (NPS)—especially depression—and low quality of life (QoL). Management of AD remains difficult, especially in the elderly. Reminiscence therapy (RT) is a well-known cognitive rehabilitation intervention that can be adopted in nursing and residential care homes to restore autobiographical memory, ameliorate NPS, and improve the QoL of people with dementia. However, the evidence-based efficacy of RT for elderly patients with AD remains to be determined. Methods: Here, we synthesized findings of randomized controlled trials (RCTs) exploring the effects of RT on cognition, depression, and QoL in elderly people with AD, according to the most recent PRISMA statement. We searched for RCTs in PubMed, Web of Science, and Cochrane Central Register of Controlled Trials, and in trial registries (i.e., clinicaltrials.gov and International Clinical Trials Registry Platform of the World Health Organization). Two review authors extracted data of interest, with cognition, depression, and QoL measures as outcomes. Results: A total of five articles were included in the final analysis. Findings globally showed that RT, both administered in individual or group sessions at least once a week for 30–35 min over a period of 12 weeks, is effective in supporting global cognition, ameliorating depression, and improving specific aspects of the QoL in elderly people with AD. Conclusions: RT has the potential to be a routine non-pharmacological therapy for elderly people with AD, thanks to its wider effects on the individual in terms of cognitive vitality and emotional status promotion, with positive implications for patient’s daily life. Despite such evidences, caution should be used in findings’ generalizability in relation to the paucity of existing RCTs with long-term follow-up.

## 1. Introduction

### 1.1. Alzheimer’s Disease (AD): Clinical Features and Assessment Tools

Because of the increasing aging population, dementia currently represents a social and health emergency in relation to progressive cognitive impairment and functional disability. AD represents the most common neurodegenerative condition characterized by the presence of extracellular senile plaques of insoluble β-amyloid peptide (Aβ) and neurofibrillary tangles composed of phosphorylated tau protein (P-tau) in the neural cytoplasm that causes dementia in the elderly. Its estimation is predicted to increase to 131.5 million by 2050, with higher proportions in the developed countries than undeveloped countries [1]. 

Up to 50% of community-dwelling older adults of 85 years or more present with AD, vascular dementia, or Lewy bodies dementia [2]. The dementia syndrome stemming from AD physiopathological processes mainly results in cognitive deterioration, functional decline, and behavioral symptoms. We recently documented how brain and cognitive reserve may play a critical role for the maintenance of mental health towards progressive deterioration in patients with mild cognitive impairment [3]. 

AD is a neurodegenerative condition characterized by an insidious onset and progressive decline in brain abilities, with memory difficulties and spatial/temporal disorientation as main features, followed by the deterioration of additional cognitive domains, such as the executive and attentional system, language, gnosis, and praxis abilities. Despite moderate psychometric properties [4], the Mini-Mental State Examination (MMSE), developed by Folstein and colleagues [5], represents the tool most widely used by clinicians to assess the progression and the severity of cognitive deterioration, with scores ranging from 0 to 30 (i.e., optimal performance). The MMSE has been applied in different settings for detecting cognitive impairment, dementia progress, and monitoring of treatment response. Scores below established cut-offs, calculated on the basis of patient’s education, may reflect dementia [6], but a more detailed assessment for each cognitive sub-domain assessed (i.e., orientation, registration, attention and calculation, recall, language, and praxis) is necessary to depict the neuropsychological profile of one patient. 

Cholinergic deficiency is widely recognized as the main hypothesis concerning cognitive defect in AD and represents the motivation for the use of cholinesterase inhibitors (Chis) that may also ameliorate neuropsychiatric symptoms (NPS) associated to this neurological condition [7]. Neuropathological changes of the brain, unmet psychological needs, physical illness, and pain concur in depicting the etiology of NPS in patients with AD. Clinical appearance includes apathy and depression, anxiety, psychotic symptoms (i.e., delusions and hallucinations), sexual and social disinhibition, agitation, aggression, aberrant motor activity, and sleep disturbances [8]. NPS have been observed in 60% to 98% of patients, especially in later dementia stages [8]. Remarkably, NPS in AD (NPS-AD) are highly prevalent and most patients exhibit one or multiple symptoms during the course of the disease that may range from mild to severe [9], especially depressed mood. Understanding brain–behavior relationship in terms of damaged neural circuits involvement may also help specialists to better target NPS-AD. In fact, specific brain regions (i.e., anterior cingulate and insula, amygdala, and frontal cortex), and neurotransmitter systems, particularly serotoninergic and dopaminergic mechanisms, have been proposed as factors underlying NPS-AD by sectorial neurobiological studies [9].

In particular, depression usually precedes or accompanies dementia onset. A recent meta-analysis determined that the prevalence of major depression disorder among older adults with AD was 14.8% [10]. Detection of NPS in AD has relied primarily on caregiver reported scales. The Neuropsychiatric Inventory (NPI) [11] represents the most relevant instrument recommended to systematically assess NPS and measure treatment effects. Particularly, the NPI use is made rapid thanks to the adoption of a screening strategy to decrease the administration time, and both the frequency and severity of each behavior can be determined. Further, the NPI reports good psychometric properties such as content and concurrent validity, intra- and interrater reliability, test–retest reliability, and internal consistency [12].

Depression and dementia remain the most frequent disorders of late life and simultaneous occurrence is quite common in the geriatric population. Thus, the application of valid and reliable screening scales, such as the Geriatric Depression Scale (GDS) and the Cornell Scale for Depression in Dementia (CSDD) may concur in diagnosis depression [13]. Specifically, the GDS is a standardized validated 30-item self-reported questionnaire originally conceived for screening depression in the non-demented elderly population, with a cut-off above the score of 9 indicating pathology. On the contrary, the CSDD was originally designed for screening depression in a population of demented elderly and consists of an interview-administered scale of 19 items relying on information from caregivers and clinician’s observation, with a cut-off score of 8 or more indicating pathology [13].

Detection of depression in AD is also important in the context of health-related quality of life (QoL). The investigation of the QoL should be used as a criterion to probe the areas in which health-care improvement is needed or as a rationale when a specific medical, nursing, or psychosocial intervention is required [14], also in the context of AD. Domains of QoL in AD include cognitive functioning, ability to perform activities of daily living and to engage in meaningful time use, social behavior, and a favorable balance between positive emotions and negative emotions [15]. Some tools to assess the QoL in older adults with AD exist, such as the Quality of Life in Alzheimer Disease (QoL-AD), a brief, 13-item measure encompassing different domains: physical health, energy, mood, living situation, memory, family, marriage, friends, self-care, ability to perform chores around the house, ability to do things for fun, and life as a whole. It is administered in an interview format to demented patients and as a questionnaire to caregivers [16]. However, its use is limited to individuals with MMSE score greater than 10 [16]. The QoL-AD measure results can be influenced by several factors such as polypharmacy, cognitive decline and functional impairment, NPS, and caregiver burden [17]. Further, the Self-Reported Quality of Life Scale (SRQoL) is another instrument that can be used to measure different dimensions of a resident’s experience on a 4-point Likert scale (from 1, never, to 4, often), i.e., comfort, functional competence, privacy, dignity, autonomy, meaningful activities, relationship, food enjoyment, spiritual well-being, security, and individuality [18], with reliability scores ranging between Cronbach’s alpha values of 0.78 and 0.85 [19]. Finally, the level of daily life activity and the QoL of older adults can be also explored using the Multi-dimensional Observation Scale for Elderly Subjects (MOSES) [20], including an evaluation of withdrawal (i.e., preferring solitude, initiating social contacts, responding to social contacts, friendship, interest in day-to-day events, keeping occupied, and helping other residents). Is has been reported that MOSES has a high reliability (i.e., internal consistency, 0.78–0.87) [21].

### 1.2. Pharmacological and Non-Pharmacological Interventions in AD

A variety of psychotropic medication, such as antipsychotics (both typical and atypical), benzodiazepines, anticonvulsants, and antidepressants are used *off-label* to treat NPS in dementia. Antipsychotic medications have the strongest evidence for efficacy. However, the treatment effect size is modest, and their use is associated with cognitive worsening and falls [22], which need to be accounted for when considering their use in elderly patients. Antidepressant medications may have some benefits in reducing agitation in dementia, but findings from randomized controlled trials (RCTs) are inconsistent [23]. Moreover, nearly a quarter of patients with dementia are prescribed benzodiazepines and sedative hypnotics [24] but very few trials really exist, and side effects are also reported [25].

In recent years, there has been growing interest in non-pharmacological interventions for people with dementia, starting from the increasing evidence that they may mitigate disease progression and psychopathological correlates as well as ameliorate QoL. Non-pharmacological interventions are, nowadays, recognized by the most relevant medical organizations (i.e., the American Geriatric Society, the American Psychiatric Society, and the American Association for Geriatric Psychiatry) as *first-line treatments* for dementia, also due to their limited potential adverse effects [26]. Non-pharmacological interventions should be continued also when pharmacotherapy is necessary [27]. 

Due to the heterogeneous nature, strategies widely vary in this area. We documented an extensive range of different interventions, as follows [28]: psychoeducational interventions and family counselling to offer information about disease and assistance for how to deal with daily problems, behavior therapy using *antecedent-behavior-consequence* (ABC) diary assessment, reality orientation therapy, cognitive stimulation for enhancing mental abilities and behavioral control monitoring, exercise, multisensory stimulation, such as music therapy and Snoezelen, bright light therapy, and aromatherapy. Simulated presence therapy, as a technique consisting of video or audiotape recordings of family members played to the person with dementia, represents another possible new way within non-pharmacological interventions [29]. We recently documented that some technological solutions have the potential for designing innovative strategies to manage AD-related symptoms, too [30].

### 1.3. Reminiscence Therapy

Among non-pharmacological interventions, RT holds a pivotal role [28]. It was developed by Butler in 1963 [31] as a dementia treatment by using a “*life-review approach*”, meaning that various memory triggers such as household objects, past photographs, and music may be used to recall autobiographical events able to enrich life experiences of elderly people. Butler believed that elderly people could share past memories through the use of stimulating prompts. In the context of AD, where new learning is difficult due to anterograde amnesia, well-rehearsed past memories are still accessible and may lead to reliving certain life experiences and to augment meaningful social interactions with others. The use of RT inside or outside the institutions delivered by psychologists and trained geriatric nurses became widely applied starting from the Eighties [32]. RT can be administered both in individual or in group sessions as a person-centered approach or to stimulate conversation and increase attention among participants, respectively [32]. RT implies the use of written/oral life histories in order to improve psychological wellbeing [33]. A Cochrane systematic review of Woods and colleagues [34] concluded that RT has some positive effects in the domains of cognition, QoL, communication, and mood of demented people. RT has been recently implemented even for family caregivers showing potential amelioration of stress [35], anxiety, and quality of relationship with the patient [36], or it can be delivered through technological devices, such as personalized digital memory books, mobile applications, and computer-aided programs [37,38]. RT has been known for its practical advantages due to a simple psychological intervention, with low application risk in people with dementia. It can be adopted in emergency departments, day-care nursing homes, long-term homes, hospitals, and even in individuals’ houses [39,40,41,42].

### 1.4. Aim of the Study

RT may be useful for elderly people with AD both because depression is common in dementia and because people with dementia typically have a better memory for past experiences then recent ones. Moreover, cognitive functioning decrement and mood alterations are strongly associated with QoL in residents of care facilities with AD. Starting from these assumptions, the objective of this systematic review was to summarize evidence from RT interventions adopted to increase global cognition, ameliorate depression, and improve QoL on elderly people with AD. 

## 2. Materials and Methods

### 2.1. Search Strategy

**Figure 1:**
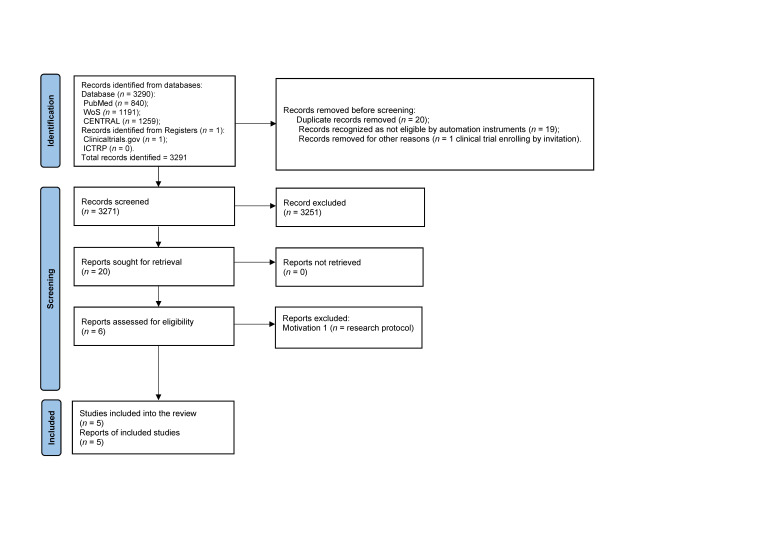
The PRIMA flow diagram.

Our systematic review adhered to the most recent *Preferred Reporting Items for Systematic Reviews and Meta-analyses* (PRISMA) *Statement* [43]. PubMed, Web of Science (WoS), and Cochrane Central Register of Controlled Trials (CENTRAL) databases for published randomized controlled trials (RCTs) were screened systematically. In addition, we searched *clinicaltrials.gov* and the *International Clinical Trials Registry Platform* of the World Health Organization (ICTRP-WHO) for any ongoing or unpublished trials. For all the searches into the databases, the following terms were used: “Alzheimer’s disease” AND “older adults” OR “elderly people” AND “reminiscence therapy” AND “randomized controlled trial” (upper time limit: 5 July 2022). We used the *EPPI-Reviewer*, developed by the *EPPI-Centre at University College London* as a web-based software program for managing extracted data and removing duplicates. A manual check in cases that needed to be deepened was further performed and results were shared between the first (D.M.C.) and the second author (G.C.) of the manuscript. Figure 1 shows the PRISMA flow diagram.

### 2.2. Study Selection Criteria

Studies from the literature search were selected if they met the following conditions: (1) patients with AD recruited on the basis of standardized international diagnostic criteria, i.e., the National Institute on Aging/Alzheimer’s Association criteria (NIA-AA) [44] or the National Institute of Neurological and Communicative Disorders and Stroke and the Alzheimer’s Disease Related Disorders Association (NICCDS-ADRA) criteria [45], (2) elderly people (>65 years of age); (3) reminiscence therapy as a unique non-pharmacological technique adopted for the intervention group; (4) RCT as study design. Exclusion criteria encompassed the following: (1) studies recruiting individuals with neurological disorders different from AD (i.e., other dementias or mild cognitive impairment) or psychiatric disorders; (2) studies recruiting healthy elderly people as participants; (3) multicomponent interventions; (4) manuscripts written in other languages than English.

### 2.3. Outcome Measures

We included studies evaluating the effects of RT on elderly people with AD measured at post-treatment, and at follow-up (typically at 6 months post-intervention evaluation) by standardized measures of cognitive functions, depression, and QoL.

### 2.4. Quality of the Studies and Assessment of Risk of Bias Evaluation

Two independent reviewers (D.M.C. and G.P.) screened the records of the searched literature. First, titles and abstracts of the retrieved records were evaluated. Second, the full-texts were carefully examined and evaluated for eligibility. Finally, the reviewers evaluated methodological criteria used by the selected RCTs (Table 1) and assessed the risk of bias according to the *Quality Assessment Tool for Quantitative Studies* [46] developed by the *Effective Public Health Practice Project* (EPHPP) (Table 2). In both cases, disagreement was discussed thanks to the support of a third reviewer (E.M.G.), until a consensus was definitely reached.

## 3. Results

### Study Selection, Evaluation, and Report

A total of 3291 records were identified through databases and trial registries searches. After removing duplicates (*n* = 20), the reviewers screened the titles and the abstract of the remaining records (*n* = 3271) and moved towards the full-text inspection of potentially eligible reports (*n* = 20). Five articles were considered eligible for the final analysis [47,48,49,50,51]. Two trials [49,50] used a quasi-randomized method of allocating participants to intervention groups and to control groups, while another one [48] consisted of a single-blind parallel-groups study (i.e., intervention, active and passive control groups). Lastly, one study [47] recruited participants with AD and vascular dementia. For the purpose of this systematic review, the authors considered the article reports of two groups separately, taking into consideration only that which related to AD patients.

The evaluation of the methodological criteria was first shown in Table 1. The assessment of risk of bias (Table 2) reported that three studies [47,48,51] were of strong quality, while two were of moderate quality [49,50]. Data extraction was performed independently and in duplicate. No meta-analysis was conducted because of the heterogeneity among the included studies, in terms of intervention types (individualized vs. group therapy administration), timing of sessions, and settings. Thus, only a qualitative synthesis of the main findings was reported (Table 3). Results globally showed that RT both administered in individual or group sessions at least once a week for 30–35 min over a period of 12 weeks is effective in supporting global cognition, ameliorating depression, and improving specific aspects of the QoL in elderly people with AD. Specifically, global cognition (measured by the MMSE), and depression (measured both by the GDS and the CSDD) improved after the RT intervention in three studies [49,50,51]. Further, the QoL (measured by the SRQoL, and the QOL-AD) improved in two studies [48,51], with a tendency towards significance in another one [47].

Characteristics of AD participants were quite similar among studies in terms of AD severity, with the baseline MMSE ranged from 13.2 ± 1.2 [48] to 15.7 ± 3.5 [49] showing a moderate cognitive deterioration, and only two studies adopting qualitative staging instruments (e.g., Clinical Dementia Rating) [47,48]. Psychotropic medication (especially antidepressants) as relevant information of patients’ treatment was poorly reported [48,49,51]. Physical disability was not described as a restricting condition for RT activities [48]. 

Individual RT was used in two studies [48,49,51] and group RT in three studies [47,50,51]. Reminiscence using a life story approach was the core practice of all interventions [47,48,49,50,51], usually delivered by materials triggering memories such as photographs, household goods, written texts, music from the past, and other things reminiscent of the past. Trained nurses and clinical psychologists ordinarily administered RT, with the presence of 1/2 activities facilitators, i.e., a group leader and a co-leader. Only in one case [48], the inclusion of patient’s relatives was permitted. No test of autobiographic memory, and memory of remote events was administered to patients in order to evaluate potential improvement after treatment. A 6-month follow-up to evaluate RT maintenance effects was only performed by two investigations [47,48].

## 4. Discussion

We performed a systematic review of RCTs analyzing RT effects on cognition, depression, and QoL of elderly patients with AD. Results were in line with previous evidence of RT effects on people with distinct types of dementia [34,41,42,52,53]. 

Pharmacological treatment for mild to moderate AD with inhibitors of acetylcholinesterase (AChEI) has shown a slight clinical improvement for global cognition [28]. Moreover, multimorbidity and polypharmacy represent interacting factors complicating the use of medication for NPS-AD in older adults [27]. Finally, frailty as a multidimensional variable mainly including physical health, comorbidities, functional status, symptoms, and health attitudes [54,55] often characterize elderly patients. Starting from these considerations, non-pharmacological interventions such as RT represent alternative strategies that can be adopted to effectively manage AD-related symptomatology. Remarkably, RT can stimulate cognitive abilities and improve general well-being. 

Our findings reported evidence for the use of RT against cognitive deterioration, depressive symptoms, and QoL, because it specifically supports people in affirming residual cognitive abilities (especially memory ones), and in enhancing self-esteem and interpersonal skills. By focusing on facts and episodes that can be retrieved from the past, a sense of mastery over cognition is experienced by patients and frustration may be limited, with positive implication for mood stability. QoL may be also improved by using personal objects as prompts in RT activities as well as the sensory systems (i.e., visual, auditory, and olfactory systems) leaving room for emotional expression. RT further allows the therapist to develop a deep understanding about the function or the meaning behind a person’s distressed behavior, in order to develop an individually tailored strategy to relieve distress. RT can also be delivered by technology and applied in different settings, such as assisted living facilities where specific training is provided for the staff, or even in familiar contexts where a cognate can be supported by an educated therapist. Formal and informal caregivers’ education on RT techniques relevant for improving coping strategies, adopting effective communication, and introducing predictable care routines, constitute an integral part of the AD management, too.

According to a previous meta-analysis [56], there is no significant difference between one-on-one and group formats in RT administration. This may be explained by the fact that RT *itself* represents an effective approach involving the recollection, the review, and the re-evaluation of personally experienced past events beyond different formats. It can aid elderly people with AD to increase self-acceptance, provide perspective, and past conflicts resolution. By comparing the results of the selected studies and verifying the convergence among them, we estimated the best duration of RT intervention in sessions of 30/35-min sessions administered *at least* once a week over a period of 12 weeks. RT is recommended by some of the most important UK authorities, such as the British Psychological Society, the National Institute for Clinical Excellence, and the Royal College of Psychiatrists [57] as an established therapy for people with dementia. A recent systematic review and meta-analysis also confirmed that RT has some benefits in reducing depression and in improving quality of life and cognition in older adults admitted to long-term care facilities with mild-to-moderate dementia [58] that constitute the clinical population target of the intervention.

Limitations of the selected studies are present, such as the lack of neuropsychological measures for better defining the cognitive profile of AD patients, especially for episodic memory assessment. Moreover, the studies did not adopt additional methods (e.g., ad hoc interviews and specific software for the analysis of qualitative data such as patients’ narrations) in order to probe the individual’s experience over the course of the intervention. Such information might improve reflections of therapists and researchers for further implementing RT protocols. 

Currently, RT is not as widely available as other non-pharmacological interventions for AD (i.e., holistic techniques, brief psychotherapies, cognitive methods, and complementary strategies) [28] and this may constitute a restraint of AD patients’ care. RT is also subordinate to the presence of trained therapists, such as clinical psychologists, and to care facilities or organizations providing multiple services for elderly people with AD.

## 5. Conclusions

AD is a progressive neurodegenerative disease with common clinical features of cognitive deterioration, neuropsychiatric manifestations (especially depressive symptoms), and low QoL that significantly impacts on sanitary assistance and social care. RT represents a useful, versatile, and cost-effective non-pharmacological intervention for managing the care of older adults suffering from AD. Particularly, RT as a psychological technique is useful in supporting global cognition, ameliorating depression, and improving some aspects of the patient’s QoL. RT administered by either individual or group (i.e., 4–5 members) sessions at least once a week for 30–35 min over a period of 12 weeks is effective in supporting global cognition, ameliorating depression, and improving specific aspects of the QoL in elderly people with AD. Despite its strengths, the paucity of RCTs with long-term follow-ups does not allow a depiction of definitive conclusions and future research is warranted to better generalize results. 

## Figures and Tables

**Table 1:** Evaluation of methodological criteria used by the RCTs examining non-pharmacological intervention for elderly people with dementia.

Selected Studies	1	2	3	4	5
Tadaka & Kanagawa, 2007 [47]	+/−	-	-	+	+
Azcurra, 2012 [48]	+/−	+	+	+	+
Van Bogaert et al., 2013 [49]	+	-	-	+	+
Duru Asiret and Kapaku, 2015 [50]	-	+/−	+	+	-
Lök et al., 2018 [51]	-	+	+	+	-

Note: (1) The diagnosis of AD is based on validated criteria; (2) Inclusion and exclusion criteria of the studies were specifically described; (3) The study reported statistics on power and sample size calculation; (4) Intervention, measurements, and outcomes are fully described; (5) Potential adverse effects are indicated and confounding variables are discussed.

**Table 2:** Assessment of risk of bias of the included RCTs.

Selected Studies	Selection Bias	Study Design	Confounders	Blinding	Data Collection Methods	Withdrawals and Dropout	Average Score
Tadaka & Kanagawa, 2007 [47]	*	***	***	***	***	**	***
Azcurra, 2012 [48]	**	***	**	***	***	***	***
Van Bogaert et al., 2013 [49]	*	***	***	*	***	***	**
Duru Aşiret & Kapaku, 2015 [50]	*	**	***	*	**	***	**
Lök et al., 2018 [51]	**	***	***	*	***	***	***

Note: * weak quality; ** moderate quality; *** strong quality.

**Table 3:** Synthesis of main findings.

References	Characteristics of AD Participants	RT Sessions, Type Administration (Individual vs. Group) and Setting	RT Intervention	Outcomes Assessment	Follow-Up	Main Findings
Tadaka & Kanagawa, 2007 [47]	24 participants assigned to the intervention group (*n* = 12; mean age, 82.5 ± 6.6) and to the control group (*n* = 12; mean age, 82.5 ± 6.6)	A group RT of 60–90 min per session, administered once a week for 8 weeks at a geriatric health facility	RT sessions started with the introduction of themes and prompts suitable to individual characteristics and life history, followed by a group discussion, and concluded by a reply of the participant to questions posed by the other ones.	MOSES MMSE	6-month follow-up	A tendency towards significance in MOSES withdrawal; no improvement in MMSE
Azcurra, 2012 [48]	135 participants assigned to the intervention group (*n* = 45; mean age, 85.3 ± 5.6), active control group receiving counselling and informal social contact (*n* = 45; mean age, 86.4 ± 4.9), and passive control group receiving unstructured social contact (*n* = 45, mean age, 85.8 ± 5.1)	An individual RT intervention delivered for 24 bi-weekly sessions lasting 1 hour each, over a period of 12 weeks at long-term nursing homes	The participants joined a group of peers and the coordinator offers memory triggers (i.e., photographs, recording, and newspaper clippings). Caregivers/relatives of the participants were sometimes included. A general discussion followed, fostering the emergence of shared concepts and reframing of patient’s initiative.	SRQoL	6-month follow-up	Significant improvement in SRQoL
Van Bogaert et al., 2013 [49]	82 participants assigned to the intervention group (*n* = 41; age in years mean/range = 83/65–98) and to the control group (*n* = 41; mean/range = 85/65–101). They were further divided into mild AD (MMSE > 18) and moderate AD (MMSE ≤ 18)	A structured individual RT intervention (i.e., “the SolCoS model”) consisting of two 45 minute sessions per week over a period of 4 weeks at three long-term facilities, two day care centres, and one psychiatric inpatients care facility	A standardized interview on participant awareness about family, home, community and life role. Memories are then evoked and recorded by audio, video, or written documents. Family, profession, holidays, and games are the topics explored. Facilitator’s role as changing agent in reminiscence process is also evaluated.	MMSE GDS CSDD	No follow-up	Improvements in MMSE for participants with moderate AD and in GDS for participants with mild and moderate AD
Duru Aşiret & Kapaku, 2015 [50]	62 participants assigned to the intervention group (*n* = 31; mean age, 81.8 ± 4.8) and to the control group (*n* = 31; mean age, 82.2 ± 5.0)	A group RT administered once a week with 30–35 minutes per session for 12 weeks at four Ministry of Family and Social Policies elderly care and rehabilitation centres	RT sessions included introduction, childhood and family life, school days, starting work and work life, a day of fun outside the home, marriage, plants and animals, infants and children, food and cooking, holidays, travel and celebrations, followed by session evaluation and closure.	MMSE GDS	No follow-up	Improvements in MMSE and in GDS
Lök et al., 2018 [51]	60 elderly participants (no mean age was specified) assigned to the intervention group (*n* = 30) and to the control group (*n* = 30)	A group RT once a week with 30–35 minutes per session for 12 weeks at a nursing home	RT sessions included discussions about childhood, festivals, memorable travel places, favourite food, important historical events, achievements and music. The participants were then encouraged to remember and share while group leader provides support to make them feeling stronger, valuable, and self-confident.	s-MMSE CSDD QOL-AD	No follow-up	Improvements in s-MMSE, CSDD and QOL-AD

Note: MOSES = Multi-dimensional Observation Scale for Elderly Subjects; MMSE: Mini-Mental State Examination; SRQoL = Self-Reported Quality of Life; SES = Social Engagement Scale; FAB = Frontal Assessment Battery; NPI = Neuropsychiatric Inventory; GDS = Geriatric Depression Scale; CSDD = Cornell Scale for Depression in Dementia; QOL-AD = Quality of Life in Alzheimer’s disease; s-MMSE = standardized MMSE.

## Data Availability

Not applicable.
